# Detection of honey bee viruses in larvae of *Vespa orientalis*

**DOI:** 10.3389/fcimb.2023.1207319

**Published:** 2023-06-23

**Authors:** Karen Power, Manuela Martano, Ernesto Ragusa, Gennaro Altamura, Paola Maiolino

**Affiliations:** ^1^ Department of Veterinary Medicine and Animal Productions, University of Naples “Federico II”, Naples, Italy; ^2^ Department of Agricultural, Food and Forest Sciences, University of Palermo, Palermo, Italy

**Keywords:** Oriental hornet, *Vespa orientalis*, honey bee, DWV, ABPV, BQCV, CBPV, SBV

## Abstract

The Oriental hornet (*Vespa orientalis*) is one of the major predators of honey bees. It has been demonstrated that adults of *V. orientalis* can harbor honey bee viruses, however the transmission route of infection is still not clear. The aim of this study was to study the possible presence of honey bee viruses in *V. orientalis* larvae and honey bees collected from the same apiary. Therefore, 29 samples of *V. orientalis* larvae and 2 pools of honey bee (*Apis mellifera*). samples were analyzed by multiplex PCR to detect the presence of six honeybee viruses: Acute Bee Paralysis Virus (ABPV), Black Queen Cell Virus (BQCV), Chronic Bee Paralysis Virus (CBPV), Deformed Wing Virus (DWV), Kashmir Bee Virus (KBV) and Sac Brood Virus (SBV). Biomolecular analysis of *V. orientalis* larvae revealed that DWV was present in 24/29 samples, SBV in 10/29, BQCV in 7/29 samples and ABPV in 5/29 samples, while no sample was found positive for CBPV or KBV. From biomolecular analysis of honey bee samples DWV was the most detected virus, followed by SBV, BQCV, ABPV. No honey bee sample was found positive for CBPV or KBV. Considering the overlapping of positivities between *V.orientalis* larvae and honey bee samples, and that *V.orientalis* larvae are fed insect proteins, preferably honey bees, we can suggest the acquisition of viral particles through the ingestion of infected bees. However, future studies are needed to confirm this hypothesis and rule out any other source of infection.

## 1 Introduction

The Oriental hornet (*Vespa orientalis* Linnaeus, 1771) is native to the southeastern Mediterranean, north-eastern and eastern Africa, the Middle East, Central Asia (Archer, 1998; Cíetkovicí, 2003), Malta and southern Italy. Due to involuntary anthropic introduction, climate change and habitat loss (Ward and Masters, 2007; Renault et al., 2018), the areal of the Oriental hornet is expanding with reports of its presence in central and northern Italy (Bressi et al., 2019; Graziani and Cianferoni, 2021), as well as in other European countries (Delmotte and Leclercq, 1980; Hernaíndez et al., 2013; Sánchez et al., 2019; Castro and del Pico, 2021; Ceccolini, 2021; Gereys et al., 2021; Zachi and Ruicănescu, 2021). *V. orientalis* is recognizable by the rusty red color of the body and the presence of characteristic yellow bands in the abdominal metasoma and yellow marks on the head between the eyes (Linnaeus, 1771; Smith-Pardo et al., 2020). The Oriental hornet is a social insect which lives in annual colonies originating from single queens. The “embryonic nests” is the primary nest which is built and cared for by the queen alone following her emergence in spring after a period of winter diapause (Matsuura, 1991). In the embryonic nest, the queen’s main role is to lay eggs, however she also performs other tasks such as building new brood comb to enlarge the nest, attending and collecting food for the developing brood. In early spring, when workers emerge from their cells, they substitute the queen in her tasks helping the colony to expand (Cappa et al., 2021). The number of colony members increases throughout spring and summer, reaching peaks of 4,000 individuals in late summer/beginning of autumn, and gradually decreases following the decrease of temperatures (Ibrahim and Mazeed, 1967). Reproduction occurs between September and December when new queens and drones emerge and mate. Subsequently, drones die while fertilized queens search for an appropriate hiding space for winter hibernation (Ishay, 1964). Feeding behavior varies according to age. Workers feed on carbohydrates which are absorbed by ingestion of fruit with soft pericarps and high sugar levels, such as figs, Indian figs, dates and grapes, nectar and honey (Ibrahim and Mazeed, 1967), while larvae are fed mainly animal proteins (flies, grasshoppers, yellowjackets and honey bees) by workers (Archer, 1998; Hernaíndez et al., 2013). Hornet larvae need to consume great amounts of proteins to appropriately develop, and honey bees appear to be among the favored protein source (Cini et al., 2018). Larvae, for their part, process the proteins ingested and return them to workers and to the queen as a drop of saliva composed of carbohydrates and free amino acids (Ishay and Ikan, 1968). *V.orientalis*, is only one of the three species of *Vespa* spp., which have been identified on the Italian territory and which have been associated to impairment of honey bee wellbeing. The European hornet (*Vespa crabro* Linnaeus, 1761) (Carpenter and Kojima, 1997) and the Asian hornet (*Vespa velutina* Lepeletier, 1836) (Bertolino et al., 2016), as the Oriental hornet, attack honey bee foragers during their foraging flights or when returning to the hive, and inhibit *A. mellifera* foraging activity (Ishay, 1964). Moreover, forager hornets can spot honey bee hives which hold the best combination of carbohydrates and proteins (Werenkraut et al., 2021) and they plunder them by stealing honey, pollen and larvae, and they then carry to the nest weakening honey bee colonies (Morse, 1978; Abrol, 1994). Inhibition of foraging activity together with scarcity of hive food storage could result in starvation of honey bees (Monceau et al., 2013; Monceau et al., 2014) and inadequate nourishment of honey bee larvae, which could develop into weak adults more susceptible to the action of pesticides and pathogens (Branchiccela et al., 2019). It should also be considered that the interaction between the honey bees and hornets can lead to transmission of pathogens, as previously reported in the review by Lester and Beggs (2019). Previous studies have already detected the presence of various honey bee pathogens in the three species of *Vespa* spp. (Nowar, 2016; Forzan et al., 2017; Mazzei et al., 2018; Mazzei et al., 2019; Highfield et al., 2020; Gabıín-Garcıía et al., 2021), and we have recently shown that adult individuals of *V. orientalis* can harbor five of the most prevalent honey bee viruses (Power et al., 2022). The prevalence of viruses in *V.orientalis* detected in our previous study reflected the epidemiological status of honey bee viral infection in apiaries across the Italian territory (Porrini et al., 2016; Bellucci et al., 2019; Bordin et al., 2022; Cilia et al., 2022), therefore, we have hypothesized that viruses could have been acquired through ingestion of infected honey bees. The aim of this study was to assess the presence of six honeybee viruses (Acute Bee Paralysis Virus (ABPV), Black Queen Cell Virus (BQCV), Deformed Wing Virus (DWV), Kashmir Bee Virus (KBV), Sac Brood Virus (SBV)) in *V. orientalis* larvae samples and honey bee (*Apis mellifera*) samples collected from the same apiary at the same time.

## 2 Methods

### 2.1 Artificial Breeding of *V. orientalis*

On the 16th of June 2022 a small nest of *V. orientalis* was identified and retrieved from a shutter box located in an apartment in Palermo (Italy). For safety reasons, adult hornets were collected using an aspirator which had been previously modified so that adult individuals could pass directly into 50 ml Falcon tubes while larvae remained in the nest. The nest was then detached from the shutter box and attached to the roof of a breeding-box using quick-setting powder gypsum, and transported to the Department of Agricultural, Food and Forest Sciences (SAAF)-University of Palermo. The breeding-box was installed in the SAAF experimental apiary approximately 20 m from where the honey bee hives are kept and it was then repopulated with the previously collected hornets. The hornets’ breeding-box was prepared and the colony managed as described by Ishay (1964) with slight modifications, particularly: side walls were realized all of the same size (40 cm x 10 cm); the bottom was made from a 2 mm thick transparent plexiglass plate; metal rails were applied along two side walls to allow the plexiglass bottom to slide; to easily close the nest, the inlet/outlet hole was modified by applying a 15 ml (conical) centrifuge tube with the bottom cut off while the top was left unmodified. The nest was monitored weekly to assess its development under artificial conditions. Forager hornets were collected when leaving the nest using a butterfly net and then marked on the thorax with a felt tip pen. Their flight activity was observed to gain information about their behavior and foraging habits.

### 2.2 Sampling

29 larvae of Oriental hornets were collected on 13th September (LVo 1-19) and 25th November 2022 (LVo 20-29) from the nest. To collect the larvae, the entrance of the nest was closed by screwing the cap to the 15 ml Falcon tube and the breeding-box was then moved a few tens of meters away from the breeding site to avoid interference of adult individuals with the collection operations. The box was then turned upside down so that the plexiglass bottom could be easily removed, and the brood cells of the nest were turned upwards, facilitating sampling of larvae from the cells. Larvae were hooked from the mouthparts, which are the toughest part and least likely to be broken, using a pair of soft plastic forceps, they were placed in 50 ml Falcon tubes and frozen at -80°C. On 25th November 2022, 10 honey bees (*Apis mellifera* Linnaeus, 1758) were also sampled from a hive located in close proximity to the hornets’ nest by scrolling from the top down a 50 ml (conical) centrifuge tube and immediately frozen at -80°C. Both honey bees and larvae samples were shipped in dried ice to the laboratory of Veterinary General Pathology and Anatomical Pathology of the Department of Veterinary Medicine and Animal Productions-University of Naples “Federico II”, where they were subjected to macroscopical examination by observation at the stereo microscope (Microscope Axioskop HBO50, Zeiss, Milan, Italy) to evaluate the presence of Varroa mites and identify possible alterations, and biomolecular analysis to detect the possible presence of honey bee viruses.

### 2.3 Biomolecular analysis

Honey bee samples were processed as two pools of 5 individuals each (HB1 and HB2) while larvae (LVo 1-29) were processed and analyzed individually. To ease homogenization, honey bees were reduced in smaller parts with sterile entomological scissors, while each larva was chopped into smaller pieces with a sterile blade after careful removal of the intestine and its content, which is composed of chitinous material not degradable by lysis buffers used in the extraction process. Homogenization was then performed with the mechanical homogenizer TissueLyser (Qiagen, Hilden, Germany). For both types of samples RNA was extracted and purified from genomic DNA using the RNeasy Plus Mini Kit (Qiagen, Hilden, Germany) and subjected to reverse transcription (RT) PCR as reported elsewhere (Power et al., 2022). A multiplex PCR thermal protocol was executed to screen for the presence of six relevant honeybee viruses (ABPV, BQCV, CBPV, DWV, KBV and SBV) according to the procedure validated by Cagirgan and Yazici (2020) and successfully employed for *V. orientalis* in our previous study (Power et al., 2022). Moreover, in each PCR reaction, one no template control (NTC) was included as negative control, while gBlocks Gene Fragments (Integrated DNA Technologies, Coralville, IA, USA) mimicking ABPV, BQCV, CBPV, DWV, KBV and SBV intended amplicons were employed as positive controls. A 150 bp segment of *V. orientalis* 28s ribosomal RNA and a fragment of *A. mellifera* β-actin were also amplified as a housekeeping genes to ensure the presence of amplifiable cDNA in each sample as previously described (Power et al., 2021; Power et al., 2022). Amplification products were then migrated by electrophoresis on 2.5% agarose gel in TAE buffer (Tris-Acetate-EDTA) along with a 100 bp molecular marker (Bioline), stained with ethidium bromide and observed under UV with the ChemiDoc gel scanner (Bio-Rad). Ethical review and approval were waived for this study, as according to the D.L. 4 March 2014 n.26, and national implementing decree following the European regulation 2010/63/UE, ethical approval is not necessary for invertebrates with the except of Cephalopoda.

## 3 Results

The colony of *V. orientalis* survived until mid December. It developed following the natural life cycle, however the nest consisted in a single “disk” of approximately 40 cm. The observation of the foraging activity of the marked hornets, has revealed that they flew to the nearby hive, captured honey bees and took them to the nest.

Macroscopical examination of samples did not reveal the presence of Varroa mites nor lesions which could have been related to viral infection. Biomolecular analysis of *V. orientalis* larvae revealed that 25/29 (86%) samples were positive for at least one virus: DWV was detected in 24/29 samples (83%), SBV in 10/29 samples (34%) BQCV in 7/29 samples (24%) and ABPV in 5/29 samples (17%), while no sample was found positive for CBPV or KBV. Moreover, several co-infections were identified in 13/29 (49%) samples. Among all, the association between DWV and SBV was the most frequent (10/13; 77%), often associated with BQCV (7/13; 54%). Three samples (3/13; 23%) revealed the presence of four different viruses, namely DWV, SBV, ABPV and BQCV. Biomolecular analysis of honey bee samples found HB1 positive for DWV, SBV and BQCV, and HB2 positive for DWV, SBV, ABPV and BQCV. No honey bee sample showed amplification of KBV or CBPV cDNAs. Amplification of 28s ribosomial RNA and β-actin in all samples confirmed the integrity of all analyzed cDNAs. For a comprehensive view of results see **Table 1**.

**Table 1:** Detection of honeybee viruses and 28s ribosomal RNA in *V.orientalis* larvae by multiplex PCR.

SAMPLE	DWV	SBV	ABPV	BQCV	KBV	CBPV	28 rRNA/β-actin
**LVo1**	**+**	–	–	–	–	–	+
**LVo2**	**+**	–	–	–	–	–	+
**LVo3**	**+**	–	–	–	–	–	+
**LVo4**	**+**	–	–	–	–	–	+
**LVo5**	**+**	–	–	–	–	–	+
**LVo6**	**+**	–	–	–	–	–	+
**LVo7**	**+**	–	–	–	–	–	+
**LVo8**	**+**	–	**+**	–	–	–	+
**LVo9**	**+**	–	**+**	–	–	–	+
**LVo10**	**+**	–	–	–	–	–	+
**LVo11**	**+**	–	–	–	–	–	+
**LVo12**	**+**	–	**+**	–	–	–	+
**LVo13**	–	–	–	–	–	–	+
**LVo14**	–	–	–	–	–	–	+
**LVo15**	**+**	–	–	–	–	–	+
**LVo16**	**-**	–	–	–	–	–	+
**LVo17**	**+**	–	–	–	–	–	+
**LVo18**	–	–	–	–	–	–	+
**LVo19**	–	–	–	–	–	–	+
**LVo20**	**+**	**+**	**+**	**+**	–	–	+
**LVo21**	**+**	**+**	–	**+**	–	–	+
**LVo22**	**+**	**+**	–	–	–	–	+
**LVo23**	**+**	**+**	**+**	**+**	–	–	+
**LVo24**	**+**	**+**	–	**+**	–	–	+
**LVo25**	**+**	**+**	–	–	–	–	+
**LVo26**	**+**	**+**	–	–	–	–	+
**LVo27**	**+**	**+**	–	–	–	–	+
**LVo28**	**+**	**+**	–	**+**	–	–	+
**LVo29**	**+**	**+**	–	–	–	–	+
**HB1**	**+**	**+**		+	–	–	+
**HB2**	**+**	**+**	+	+	–	–	+

Sample names and viruses acronyms are indicated (LVo1-29, V.orientalis larvae samples; HB1-HB2, honey bee samples; DWV, Deformed Wing Virus; SBV, SacBrood Virus; ABPV, Acute Paralysis Virus; BQCV, Black Queen Cell Virus; KBV, Kashmir Bee Virus; Chronic Bee Paralysis Virus; + positive sample; -negative sample).

## 4 Discussion

Social hornets pertaining to the *Vespa* genus are spreading across the Italian territory causing great damage to the beekeeping field by predating honey bee foragers and larvae, pillaging honey bee food supplies and participating to the diffusion of pathogens. Climate change and the rise of temperatures have encouraged settlement of *V. orientalis* outside its natural habitat and have promoted the development of a greater number of colonies (Hulme, 2017). The spread of *V. orientalis* leads to a novel combination of interacting species, which could lead to interspecific transmission of pathogens from a maintenance species (reservoir) to an incidental or non-maintenance species (Power and Mitchell, 2004; Nanetti et al., 2021). Moreover, *V. orientalis*, as a polyphagous individual, can prey on a great variety of insect species and take up sugars from nectar and fruits which have been previously visited by other insects, increasing the spectrum of interactions and the connected risk of pathogen transmission. It has been suggested that pathogen transmission occurs more easily from farmed species, such as honey bees and commercial bumblebees, to wild species; indeed, spillover phenomena of viruses from honey bees have been reported in many other arthropods (Eyer et al., 2009; Martin and Brettell, 2019; Gusachenko et al., 2020; Payne et al., 2020), including *Vespa* spp. Previous studies have described the presence of replicative forms of DWV in *V. crabro* (Forzan et al., 2017) and in *V. velutina* (Mazzei et al., 2018), which can also harbor BQCV and KBV (Mazzei et al., 2019). In *V. orientalis* the presence of *Paenibacillus larvae*, causative agent of the American Foulbrood has already been detected (Nowar, 2016), suggesting a possible role of the Oriental hornet in spreading the disease. In our earlier study (Power et al., 2022) we have detected the presence of honey bee viruses in 30 samples of *V. orientalis* collected in the Campania region and DWV was the most prevalent virus followed by ABPV, BQCV, KBV and SBV, meanwhile no sample was found positive for CBPV. Our former results reflected previous data regarding the most frequently found viruses in honey bees and non-honey bee arthropods, where DWV is the most detected, BQCV and ABPV are highly prevalent, and SBV and KBV can be detected with lower frequency (Porrini et al., 2016; Bellucci et al., 2019; Nanetti et al., 2021). In the present study, larvae samples showed positivity for DWV (83%), SBV (34%) BQCV (24%), and ABPV (17%). While the higher prevalence of DWV is in line with our previous results (Power et al., 2022), such a high prevalence of SBV is in contrast with our previous findings. However, this could be due to the fact that samples analyzed in the present study were collected from a different region, and that, conversely to the Campania region, SBV could be highly prevalent in Sicily as occurs in other areas of the Italian territory, such as Veneto (Bordin et al., 2022). Interestingly, virus detected in *V. orientalis* larvae samples collected on the 25th November 2022 (LVo 20-29) matched perfectly with that of honey bee samples (HB1 and HB2) collected on the same date from the same apiary. It is well known that honey bee viruses can be transmitted to other Hyemenoptera *via* vertical or horizontal routes (Yañez et al., 2020), therefore in larval samples of *V. orientalis* infection could have occurred from the queen to the eggs or through ingestion of contaminated food. However, viral prevalence appears different in samples collected on the 13th September (LVo 1-19) from those collected on 25th November 2022 (LVo 20-29), and only the latter showed amplification of DWV, SBV and BQCV cDNAs. Considering that all the analysed samples (LVo1-LVo29) pertain to the same generation, being all the individuals sisters and daughters of the same mother, if transmission had occurred *via* vertical pathway at least one of the samples collected in September, should have been infected with SBV and BQCV. Therefore, we tend to exclude vertical transmission of viral particles from mother to offspring. In honey bees, viral infections are often mediated by *Varroa destructor* (Shen et al., 2005; Santillaín-Galicia et al., 2010) as well as by the ingestion of contaminated pollen, nectar, bee bread, royal jelly and honey (Mazzei et al., 2014; Schittny et al., 2020). In *V. velutina*, mites belonging to the genus Varroa have been found attached to the lateral–ventral part of the abdomen with a prevalence of 1.75% (Sánchez and Arias, 2021). However no Varroa mites have been found in nests (Chauzat et al., 2015) and, to the best of our knowledge, no study has detected it in the life cycle of *V. velutina* (Rodríguez-Flores et al., 2022). To date, also in *V. orientalis* no reporting of infestation with *V. destructor* or other Varroa mites has been released, and no varroa mites were detected either in our hornet nest or during the macroscopic examination of the larval samples. However, in order to exclude without any doubts a possible transmission mediated by Varroa, an extensive survey on the presence of this parasites in *V. orientalis* should be performed. According to the information known about the biology of *V. orientalis* (Ishay, 1964; Ibrahim and Mazeed, 1967; Ishay and Ikan, 1968; Hernaíndez et al., 2013), larvae are fed by workers protein sources, mainly of animal origin. The observation of the foraging activity of the marked hornets, has revealed that they flew to the nearby hive, captured honey bees and took them to the nest, suggesting feeding of larvae with those honey bees, at least in part. The biology of the insect and the overlapping of positivities with honey bee samples could make us lean towards the acquisition of viral particles through the ingestion of infected bees. However, future studies are needed to confirm this hypothesis and rule out any other source of infection. Also, in the present study the replicative capability of the viruses was not evaluated, therefore, it is still not clear if these viruses represent real infections or if *V. orientalis* is an “incidental host” which acquired viruses passively. Future evaluation of replication, together with localization of viral particles with histological techniques, could help in better understanding the pathogenicity of honey bee viruses in *V. orientalis.*

## Data availability statement

The raw data supporting the conclusions of this article will be made available by the authors, without undue reservation.

## Author contributions

Conceptualization, KP and PM. Sampling, ER. Methodology, GA. Validation, MM and PM. Writing—Original Draft Preparation, KP. Writing—Review and Editing, KP and MM. Supervision, PM. All authors read and approved the final version of this manuscript.
